# Problematic mobile phone use: Validity and reliability of the Problematic Use of Mobile Phone (PUMP) Scale in a German sample

**DOI:** 10.1016/j.abrep.2020.100297

**Published:** 2020-08-15

**Authors:** Katharina Graben, Bettina K. Doering, Franziska Jeromin, Antonia Barke

**Affiliations:** a Philipps-University, Faculty of Psychology, Gutenbergstrasse 18, D-35032 Marburg, Germany; b Catholic University Eichstaett-Ingolstadt, Faculty of Psychology, Ostenstrasse 25, D-85072 Ingolstadt, Germany

**Keywords:** Addiction, Problematic use, PUMP, Validity, Reliability, Mobile phone

## Abstract

**Background:**

Mobile phones are ubiquitous in everyday life. Scientific studies on the problematic use of mobile phones have given initial indications of negative consequences, such as increased depression and anxiety rates and reduced sleep quality. The Problematic Use of Mobile Phone (PUMP) scale is a well evaluated, 20-item questionnaire, but a German version of the scale is still lacking.

**Method:**

An online sample (n = 723, age 27.8 ± 11.2 years, 25.2% men) completed a German translation of the PUMP scale (PUMP-D). We conducted standard item analyses and calculated internal consistency and retest reliability. An exploratory (EFA) and a confirmatory factor analysis (CFA) were conducted using a random split of the sample, and correlations with the self-estimated time of mobile phone usage and a global self-rating of patterns of problematic use were computed. Additionally, a second sample (n = 256, age 25.0 ± 8.8 years, 34.0% men) completed the paper version of the PUMP-D scale twice to determine the 14-day retest reliability.

**Results:**

The item-total correlations ranged from *r* = 0.35 (p < 0.001) to *r* = 0.75 (p < .001). The internal consistency was α = 0.90. The self-estimated time of usage correlated with the total value of the PUMP-D scale at *r* = 0.50 (p < .001). The EFA resulted in a single factor, which explained 36% of the variance. The CFA of the showed a moderate fit. The two-week retest reliability in the second sample was *r*_tt_ = 0.87 (p < .001).

**Discussion:**

The German translation of the PUMP-D demonstrated a single factor structure, good psychometric properties and can be used in further research.

## Highlights

• The German PUMP scale demonstrated very good reliability and validity and a high test-retest reliability.
• Reasonable stability of the construct “problematic mobile phone use” was shown.
• Problematic mobile phone use is a relevant issue in Germany.

## 1 Introduction

As the first thing in the morning, the last thing in the evening, over half of the American people look at their mobile phone (Lookout, 2012). Over the past 20 years, the percentage of German households possessing a mobile phone has risen to 96.7% (Destatis, 2018), and 78% of Germans own at least one mobile phone (Ametsreiter, 2017). A vast number of applications is supplied for use on mobile phones: in April 2019, the market-leading app store Google play provided nearly 2.6 million applications (AppBrain, 2019). Beside the benefits of mobile phones, especially smartphones) (e.g. accessing the Internet, maps and email on the go) many studies found associations between the amount of mobile phone use and mental health issues, like depression (Demirci et al., 2015, Harwood et al., 2014), anxiety (Demirci et al., 2015, Elhai et al., 2016, Harwood et al., 2014), chronic stress (Augner & Hacker, 2012); poor sleep quality (Demirci et al., 2015, Liu et al., 2017) and low self-esteem (Bianchi and Phillips, 2005, Ehrenberg et al., 2008, Takao et al., 2009, Yang et al., 2010). Even concepts connected with the problematic use of mobile phones were invented, such as “nomophobia” (=*no mo*bile phone *phobia*), the fear of being without your mobile phone (Bragazzi and Del Puente, 2014, Lucia et al., 2014, Yildirim and Correia, 2015).

The question of whether excessive mobile phone use should be considered a behavioural addiction along the lines of pathological gambling is being discussed (Billieux, Maurage, Lopez-Fernandez, Kuss, & Griffiths, 2015). The criteria for gambling disorders in the Diagnostic and Statistical Manual of Mental Disorders (DSM-5; American Psychiatric Association, 2013) are difficult to transfer to problematic mobile phone use, because they are strongly connected with spending money on the gambling and incurring debts. Since the advent of flat rates, this is no longer an issue with problematic mobile phone use. However, if one considers these criteria (*mutatis mutandis*) as expressions of serious negative consequences, they may be applicable to other behaviours, such as excessive mobile phone use, as well. The remaining criteria for gambling disorders may apply more directly to mobile phone use: 2. Is restless or irritable when attempting to cut down or stop ----, 3. Has made repeated unsuccessful efforts to control, cut back, or stop ----, 4. Is often preoccupied with ----, 5. Often ---- when feeling distressed (e.g., helpless, guilty, anxious, depressed), 7. Lies to conceal the extent of involvement with ----, 8. Has jeopardized or lost a significant relationship, job, or educational or career opportunity due to ----. Whether addiction criteria are useable for problematic mobile phone use or even “mobile phone addiction” is highly controversial. For example, Billieux et al. (2015) argue that some addiction criteria are difficult to transfer to mobile phone use. For example, “withdrawal” may be due to a variety of factors that would not normally be considered relevant to addiction, such as anxiety (ability to call an ambulance at any time), dependent traits (constant contact with some other person), etc. Even substance-related addictions are not as homogenous with regard to the criteria as one might assume: Cocaine, for example, is highly addictive but causes almost no physical withdrawal (Gawin, 1991).

The parallels of symptoms for problematic mobile phone use and gambling disorder suggest that mobile phone use may become a behavioural addiction and instruments for its assessment are needed. There are various questionnaires referring to problematic mobile phone use, mobile phone addiction or related constructs (e.g. Nomophobia, Compulsive Cell Phone Use or Text Message Addiction) but most of them are little used and barely validated. In the following, we will address the most commonly used instruments:

In the **Mobile Phone Problem Use Scale (MPPUS;** Bianchi & Phillips, 2005), 27 statements have to be rated on a 5-point Likert scale. The items are based on literature about behavioural addictions and assumed social aspects of mobile phone use. Cronbach’s alpha for the original scale (27 items) was reported as α = 0.91. Retest data and factor analyses are not available. A German short version with ten items (MMPUS-10) was created (Foerster, Roser, Schoeni, & Röösli, 2015) and achieved α = 0.85. Foerster and colleagues calculated a relatively low one-year retest reliability for this short version of *r*_tt_ = 0.40. Some aspects of the MPPUS are problematic. Firstly, several items measure not just problematic user patterns of the person answering the questionnaire, but also relate to their social environment, such as “All my friends own a mobile phone” and “My friends don’t like it when my mobile phone is switched off”. A second problem arises from the way that mobile phone use and the circumstances surrounding it have undergone changes since the development of the questionnaire in 2005: the item “I have received mobile phone bills I could not afford to pay”, for example, seems less relevant today.

The **Smartphone Addiction Scale (SAS;** Kwon et al., 2013) contains 33 items, which are rated on a 6-point Likert scale. The SAS consists of six subscales: daily-life disturbance, disturbance of reality testing, positive anticipation, withdrawal, cyberspace-oriented relationship, overuse, and tolerance. Cronbach’s α = 0.97 is reported, and a German version is available (Haug et al., 2015). The SAS is a modified version of a Korean self-diagnostic program for Internet addiction (K-Scale; (Kim, Kim, Park, & Lee, 2002). Six factors (previously 7 factors were assumed) were found in a factor analysis, explaining 60.99% variance, but 15 questions failed to fit at any of the factors and were excluded from the questionnaire – resulting in 33 items (of previously 48). The questionnaire’s usability is limited by its length. Additionally, some of the items assess indirect indicators of excessive mobile phone use that may be caused by factors other than patterns of use. For example, the item “Feeling pain in the wrists or at the back of the neck while using a smartphone” may be due to other health problems, and “My fully charged battery does not last for one whole day” may depend on technical aspects of the mobile phone.

The **Smartphone Addiction Inventory (SPAI;** Lin et al., 2014) consists of 26 items, which are rated on a 4-point Likert scale. The items were modified versions of the items taken from the Chen Internet Addiction Scale (CIAS; Chen, Weng, Su, Wu, & Yang, 2003). Four factors were extracted (compulsive behavior, functional impairment, withdrawal, and tolerance), explaining 57.28% of the variance. A two week test–retest reliability resulted in 0.80–0.91 and Cronbach's α = 0.94.

With 20 items, the **Problematic Use of Mobile Phones (PUMP)** scale (Merlo, Stone, & Bibbey, 2013) is the shortest instrument. The items were inspired by the criteria for substance dependence (see Table 1) in the DSM-5 (American Psychiatric Association, 2013). However, the PUMP scale does not claim that overuse of mobile phones is an addiction. The authors also generated items from a review of measures assessing consequences of excessive Internet use and informal interviews with several self-identified “cell phone addicts”. The final scale consists of statements formulating possible thoughts, feelings, and behaviours related to problematic smart phone use, such as: “When I stop using my cell phone, I get moody and irritable”. The extent to which each of these statements fits with the respondent’s self-perception has to be rated on a 5-point scale, from 1 = “strongly disagree” to 5 = “strongly agree”. The PUMP scale demonstrated very good internal consistency, with α = 0.94. A factor analysis supported a one-factor solution, with factor loadings for all items ʎ ≥ 0.48, which explained 49.05% of the variance.

**Table 1:** DSM-5 criteria for substance dependence and the related items of the PUMP Scale as described by the original authors (Merlo et al., 2013).

DSM-5 criteria for substance dependence	**Item in PUMP**
(1) More use than was intended	**5, 6**
(2) desire/unsuccessful efforts to cut down/control use	**/**
(3) spending a great deal of time	**7, 8**
(4) craving	**9, 10**
(5) resulting in a failure to fulfill major role obligations	**15, 16**
(6) continued use despite social/interpersonal problems	**19, 20**
(7) reduction or giving up of activities	**11, 12**
(8) use in situations in which it is physically hazardous	**17, 18**
(9) continued use despite physical/psychological consequences	**13, 14**
(10) tolerance	**1, 2**
(11) withdrawal	**3, 4**

### 1.1 Objective

In English, the PUMP has emerged as a useful and brief scale for assessing problematic smart phone use. Starting from a theoretical basis (DSM-5) it addresses mobile phones, including smartphones and not web-enabled cell phones. A German version as well as retest data and further studies regarding its factor structure are still lacking. For this reason, we translated the PUMP scale into German and investigated its reliability, including its two-week retest reliability, and factor structure and additional indicators of validity.

## 2 Method

### 2.1 Ethics

The study was conducted in accordance with the Declaration of Helsinki and approved by the internal review board of *blinded for the review* University (2016–16 k). All participants received full information about the study and provided informed consent.

### 2.2 First study

The first study was conducted for a general psychometric evaluation of PUMP-D.

#### 2.2.1 Procedure and participants

For the first (and main) study, the questionnaires were implemented into the online survey software *unipark* (Questback GmbH, Köln, www.unipark.de), and for recruitment, it was advertised online in multiple Facebook groups and via email at the *blinded for the review* University (see Table 2). After the informed consent, the participants provided demographic information and information regarding their mobile phone use and filled in various questionnaires, including the PUMP-D (see below). For complete participation in the first survey, we offered the chance to win one of two gift vouchers for a popular online store (voucher value €25).

**Table 2:** Systematic study design overview containing format, aim of the study, type, sample size, used instruments and conducted analyses.

	Study 1	Study 2
Format	Online	Paper/pencil
Aim	General psychometric evaluation of PUMP-D	Ascertaining 2-week retest reliability of PUMP-D
Type	Crossectional	Repeated measurements two weeks apart
Sample size	n = 723	n = 256
Instruments	Demographic information PUMP-D MPPUS CES-D SES	Demographic information PUMP-D
Analyses	Standard item analyses Reliability: MacDonald‘s Omega Guttman‘s Split Half Factor structure: Random split of sample: Subsample 1: EFA Subsample 2: CFA Correlations: MMPUS CES-D SES Usage time Age	Retest reliability

Inclusion criteria for participation were: age over 18 years, possession of a mobile phone, and German as a first language. A total of 958 participants provided informed consent; of these, 829 fulfilled the inclusion criteria and were eligible to participate. Of the eligible participants, 105 did not complete the questionnaire until the end of the PUMP-D scale and were excluded from further analyses. Because of a systematic answer pattern, one further person had to be excluded. The remaining 723 participants were aged 27.8 ± 11.2 years, and the percentage of women was 74.3%.

#### 2.2.2 Material

##### 2.2.2.1 Demographic information and mobile phone use

We asked for the participants’ sex, age, education level, civil status, and hours of mobile phone usage per day.

##### 2.2.2.2 Problematic mobile phone use

The PUMP scale was translated into German and then translated back into English (by *blinded for the review*) following the guidelines of Beaton (Beaton, Bombardier, Guillemin, & Ferraz, 2000). At first assessment, the participants completed the PUMP-D and the Mobile Phone Problem Use Scale (MPPUS; Foerster et al., 2015). The MPPUS was used to address the construct validity of the PUMP-D.

In addition, the participants answered a single-item question on whether they regarded their own mobile phone usage as problematic (0 = no, 1 = rather not, 2 = rather yes, 3 = yes).

##### 2.2.2.3 Depressive symptoms

As a measure for depressive symptoms, we used the 10-item Centre for Epidemiological Studies Depression Scale (CES-D-10; Andresen, Malmgren, Carter, & Patrick, 1994) (translation by Hautzinger (1988)). The items assess depressive symptoms in the past week, and participants are asked to state the symptoms’ frequencies on a 4-point Likert scale, ranging from “(0) rarely or none of the time” to “(3) all of the time”. The CES-D-10 has shown to have good psychometric properties with Cronbach’s α between 0.78 and 0.89 (Björgvinsson et al., 2013, Boey, 1999).

##### 2.2.2.4 Self-esteem

As a measure for self-esteem, we used the Rosenberg Self-esteem Scale (SES), which is one of the best-established scales for this construct and has good psychometric properties (Blascovich & Tomaka, 1991). The SES is a unidimensional 10-item scale. The participants rate their agreement with positive and negative feelings about themselves on a 4-point Likert scale (from “strongly agree” to “strongly disagree”). We used a validated German version with Cronbach’s α = 0.84 (von Collani & Herzberg, 2003).

#### 2.2.3 Data analysis

Standard item analyses were calculated to determine mean item scores, standard deviations, item-difficulties, item-total correlations (with the item itself excluded from the total score), and internal consistency when the item is removed. As measures of reliability, McDonald’s Omega and Guttmans split-half coefficients were computed. Missing data were excluded on a case-wise basis. To investigate the factor structure, the sample was randomly split into two subsamples in order to conduct an EFA and a CFA in two independent samples and their equivalence with regard to gender, age and PUMP-D scores compared with *X*^2^ tests and independent *t* tests, respectively. For the EFA, the adequacy of the data for factor analysis was tested with the Kaiser-Meyer-Olkin (KMO) measure of sampling adequacy and Bartlett’s test of sphericity. The number of components to be extracted was determined through Horn’s parallel analysis. For the CFA, we tested three 1-factor models allowing for different covariations (Model 1): no covariances, (Model 2): such covariances as suggested by the original authors’ allocation of items to DSM criteria and (Model 3): covariances based on item content. The following fit measures are reported: *X^2^*/df, Root mean square error of approximation (RMSEA); Comparative fit index (CFI), Standardized root mean square residual (SRMR) and the Akaike information criterion (AIC).

All analyses were computed with SPSS version 21.0.0 (IBM, Meadville, USA) and the CFA was calculated using SPSS AMOS 26.0.0.

### 2.3 Second study

The second study was conducted to gather data for retest-reliability (see Table 2).

#### 2.3.1 Procedure and participants

The participants were recruited in regularly occurring university classes in *blinded for the review* University. The procedure was adapted to a hard copy format to facilitate the assessment and re-assessment of the students when they attended weekly lectures. The questionnaire was distributed in different lectures two weeks apart and collected by research assistants. Self-generated codes permitted linking individual questionnaires across the assessments. The questionnaire contained only a subset of instruments and questions necessary to evaluate the retest reliability, namely, the demographic questions and the PUMP-D. Participants who completed both measurements could win one of two gift vouchers for a popular online store (voucher value €25). In the second study, 517 students completed the questionnaire at the first measurement time (t_0_). Nearly half of them (n = 256) participated again at t_1_ and were included in the retest analyses. They were aged 24.8 ± 8.8 years, and the percentage of women was 65.20%.

#### 2.3.2 Material

##### 2.3.2.1 Demographic information and mobile phone use

We asked for the participants’ sex, age, education level and civil status.

##### 2.3.2.2 Problematic mobile phone use

The PUMP-D scale as described in Section 2.2.2 was used.

#### 2.3.3 Data analyses

The 14-day retest reliability (Pearson correlation coefficient) was calculated. All analyses were computed with SPSS version 21.0.0 (IBM, Meadville, USA).

## 3 Results

### 3.1 First study

#### 3.1.1 Participants’ characteristics

In the first study, the mean self-reported time of mobile phone use per day was 2.8 ± 1.9 h. The vast majority, 692 persons, owned a smartphone, and the other participants (31) owned a conventional mobile phone. The mean PUMP-D score was 37.5 ± 12.6. Nearly one-third of participants rated their own user patterns as “problematic” (4.6%) or “rather problematic” (26.7%). Participants were in the mean 24.8 years old (±8.8) and 74.3% were women.

#### 3.1.2 Item analysis

Item analyses were conducted in the sample of the first study. Item difficulties varied from *p*i = 0.07 (item 19) to *p*i = 0.57 (item 13), with a mean item difficulty of *p*i = 0.31. The item-total correlations of the items with the total score ranged from *r*_itc_ = 0.35, p < .001 (item 18) to *r*_itc_ = 0.75, p < .001 (item 5); the mean item-total correlation was *r*_itc_ = 0.59 (see Table 3).

**Table 3:** Item means and standard deviations, item difficulties, item-total correlations, McDonald's ω for the subscales if the item was removed for the total sample (n = 723) and factor loadings for the EFA [n = 362] in the first study.

Item	M	SD	Difficulty	Item-total correlation	ω if removed	Factor loadings(EFA)
*1. When I decrease the amount of time spent using my cell phone I feel less satisfied.*	1.6	0.8	0.14	0.56	0.900	0.57
*2. I need more time using my cell phone to feel satisfied than I used to need.*	1.5	0.7	0.12	0.58	0.900	0.61
*3. When I stop using my cell phone, I get moody and irritable.*	1.3	0.6	0.26	0.55	0.900	0.59
*4. It would be very difficult, emotionally, to give up my cell phone.*	2.7	1.4	0.54	0.58	0.901	0.56
*5. The amount of time I spend using my cell phone keeps me from doing other important work.*	2.4	1.2	0.48	0.75	0.896	0.75
*6. I have thought in the past that it is not normal to spend as much time using a cell phone as I do.*	2.1	1.3	0.42	0.69	0.898	0.67
*7. I think I might be spending too much time using my cell phone.*	2.6	1.5	0.52	0.75	0.896	0.73
*8. People tell me I spend too much time using my cell phone.*	1.5	0.9	0.12	0.59	0.900	0.58
*9. When I am not using my cell phone, I am thinking about using it or planning the next time I can use it.*	1.6	0.9	0.32	0.64	0.898	0.65
*10. I feel anxious if I have not received a call or message in some time.*	1.6	1.0	0.16	0.52	0.902	0.53
*11. I have ignored the people I’m with in order to use my cell phone.*	1.8	1.0	0.37	0.58	. 900	0.58
*12. I have used my cell phone when I knew I should be doing work/schoolwork.*	3.1	1.4	0.53	0.75	0.897	0.73
*13. I have used my cell phone when I knew I should be sleeping.*	2.9	1.5	0.57	0.69	0.899	0.67
*14. When I stop using my cell phone because it is interfering with my life, I usually return to it.*	2.1	1.2	0.43	0.75	0.896	0.76
*15. I have gotten into trouble at work or school because of my cell phone use.*	1.2	0.6	0.24	0.48	0.902	0.50
*16. At times, I find myself using my cell phone instead of spending time with people who are important to me and want to spend time with me.*	1.6	1.0	0.14	0.56	0.901	0.54
*17. I have used my cell phone when I knew it was dangerous to do so.*	2.0	1.3	0.25	0.50	0.903	0.44
*18. I have almost caused an accident because of my cell phone use.*	1.3	0.8	0.26	0.35	0.906	0.35
*19. My cell phone use has caused me problems in a relationship.*	1.3	0.7	0.07	0.41	0.904	0.42
*20. I have continued to use my cell phone even when someone asked me to stop.*	1.3	0.8	0.26	0.48	0.902	0.49

#### 3.1.3 Reliability

In the first study, the internal consistency of the questionnaire was McDonald’s Omega ω = 0.91 and the consistency would have benefitted only marginally (+0.001) from removing item 18. The split-half reliability (Guttman’s split-half coefficient) was α = 0.87.

#### 3.1.4 Validity

##### 3.1.4.1 Factor analyses

According to the KMO criterion, sampling adequacy in the first study was excellent (KMO = 0.920), and Bartlett’s test for sphericity showed the correlation matrix to be suitable for factor analysis (χ^2^ = 5796.71, *df* = 190, *p* < .001).

For conducting an exploratory and a confirmatory factor analyses the sample was randomly split into two groups (n = 362, n = 361). The subsamples did not differ with regard to age (*t*(712) = −0.14, p = .89), gender (χ^2^(4) = 4.86, *p* = .301), hours of mobile phone use per day (*t*(718) = −0.01, *p* = .99) or PUMP-D-scores (*t*(721) = −51, *p* = .61).

##### 3.1.4.2 Exploratory factor analyses

A Maximum Likelihood estimation was calculated for one half of the sample (n = 362). The first factor explained 35.58% of the variance, and the factor loadings show all positive loadings between 0.35 ≤ ʎ ≤ 0.76 (see Table 3). The eigenvalue of factor 1 is 7.12, further factors 2 to 4 show much lower eigenvalues (1.86, 1.44, 1.01). However, the scree test and Horn’s parallel analysis supported a single factor solution.

##### 3.1.4.3 Confirmatory factor analyses

**Fig. 1:**
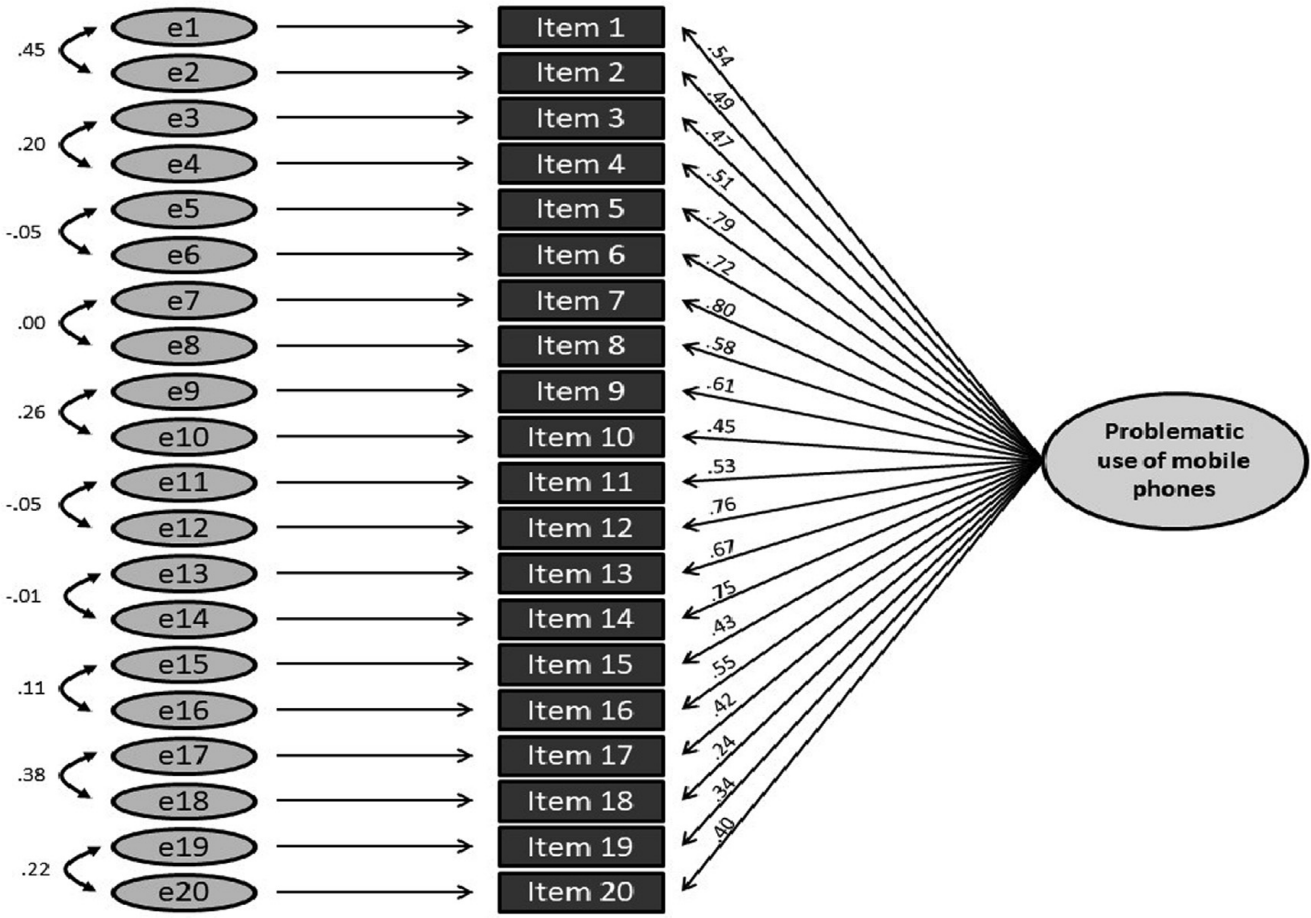
Path diagram for the confirmatory factor analysis of the PUMP-D for Model 2 with intercorrelated items for each addiction criterion, showing standardised path coefficients. All path coefficients are significant at p < .001.

**Fig. 2:**
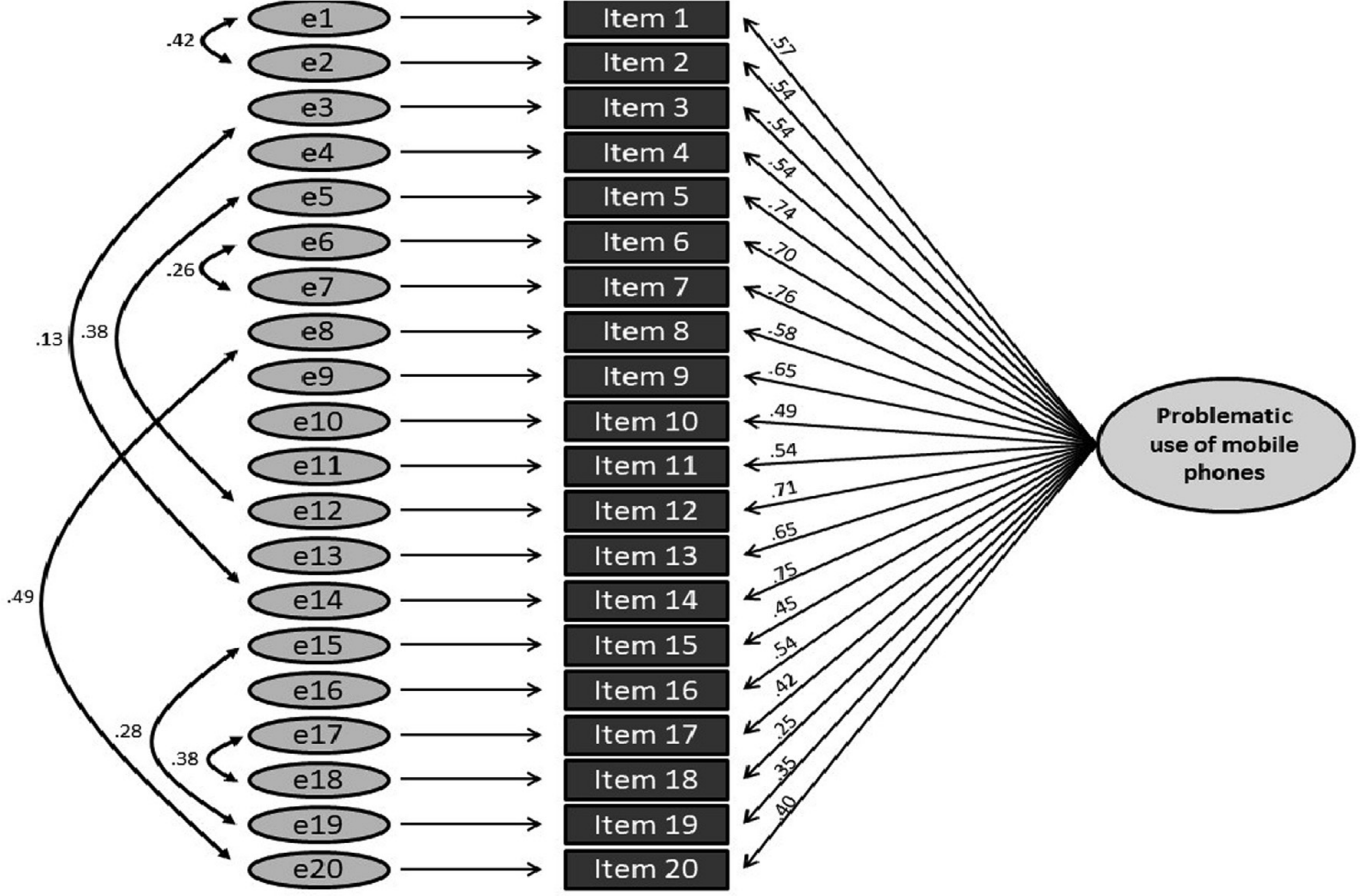
Path diagram for the confirmatory factor analysis of the PUMP-D for Model 3 with intercorrelated items, showing standardised path coefficients. All path coefficients are significant at p < .001.

In order to test the previously established one-factor structure, a confirmatory factor analysis was carried out for the second half of the sample (n = 361). We examined three one-factors models: One without correlated items (model 1), one (model 2, see Fig. 1) with allowing items to correlate according to the original authors’ allocation to criteria (cf. Table 1) and model 3, in which the items were allowed to covary according to item content (Fig. 2) Regression weights were significant with p < .001 in all models. Inspection of the fit indices (see Table 4) indicated a progressively better fit from model 1 to 3.

**Table 4:** Fit indices for PUMP-D tested in the confirmatory factor analysis (n = 360).

Model	*X ^2^*	df	*p*	*X ^2^*/df	RMSEA [90% CI]	SRMR	CFI	AIC
1	894.61	170	<0.001	5.26	0.109 [0.102; 0.116]	0.0806	0.753	974.61
2	707.12	160	<0.001	4.42	0.098 [0.090; 0.105]	0.0760	0.813	807.12
3	573.30	163	<0.001	3.52	0.084 [0.076; 0.091]	0.0668	0.860	667.30

*Notes:* Model 1: 1-factor model without any covariances; Model 2: 1-factor model with covariances as suggested by original authors’ allocation of items to DSM criteria; Model 3: 1-factor model on the basis of item content; RMSEA: Root mean square error of approximation; SRMR: Standardised root mean residual; CFI Comparative fit index,;AIC Akaike information criterion.

#### 3.1.5 Correlations

The PUMP-D scale showed a high positive correlation with self-reported time of mobile phone use per day (r = 0.50, p < .001) and self-rated problematic user patterns (*r = 0.*65, p < .001). The correlation with the MPPUS was *r* = 0.87, p < .001.

#### 3.1.6 Exploratory analyses

Considering demographic variables, no significant correlation was found between the PUMP-D and sex. A negative correlation was identified between the PUMP-D and age (*r* = −0.37, p < .001). Participants with more problematic mobile phone use had lower self-esteem (*r* = −0.25, p < .001) and more depressive symptoms (*r* = 0.38, p < .001). The self-rated problematic user patterns also correlated negatively with the SES (*r* = −0.14, p < .001) and positively with the CES-D-10 (*r* = 0.25, p < .001).

### 3.2 Second study

#### 3.2.1 Participants’ characteristics

In the second study, participants were in the mean 27.8 years old (±11.2) and 65.2% were women. All but two people owned a smartphone (the other two owned a conventional mobile phone instead). The mean PUMP-D score was 43.5 ± 12.1.

#### 3.2.2 Reliability

The Pearson correlation coefficient showed a 14-day retest reliability of *r* = 0.87, p < .001.

## 4 Discussion

The German Version of the PUMP (PUMP-D) scale demonstrated very good reliability and validity in a large online sample (first study) and a high test–retest reliability in a smaller, hard-copy study (second study).

Overall, the item-total correlations with the total scale were medium (>0.30) to high (>0.50). Items were mostly comparatively difficult, that is to say, not many respondents endorsed them. This is common in questionnaires asking for emotions and behaviours that only a minority of respondents engage in, such as addictive behaviours or problematic user patterns. The most difficult item, which in the context of our measurements means the item least endorsed by participants) was item 19 (“My cell phone use has caused me problems in a relationship”) with a difficulty of 0.07. Considering the item’s content, this appears plausible: the extent of problematic use patterns has to be serious to engender problems in a relationship. In a German study (Bitkom, 2017) more than half of the respondents aged 18 to 34 indicated that they looked at their mobile phone 26 or more times per day; interestingly, 40% of younger respondents (18–24) said they looked at their mobile phone more than 50 times per day. Frequently checking one’s mobile phone therefore seems to be age-dependent. The mean age of our participants was 27.8 ± 11.2 years, so it can be speculated that frequent mobile phone use is considered quite normal in relationships in their age group.

The least difficult item (0.57) was item 13 (“I have used my cell phone when I knew I should be sleeping”), this indicated that many people agree with this item even if they may not show problematic use patterns. This is supported by a study from Lemola (Lemola, Perkinson-Gloor, Brand, Dewald-Kaufmann, & Grob, 2014), which demonstrated that even just owning a smartphone (in this study only mobile phone with internet access were included) correlates with later bedtimes and more electronic media use in bed before sleep.

Regarding reliability, the internal consistency of the PUMP-D was excellent, with ω = 0.91, and comparable to the original version, for which Cronbach’s α = 0.94 was reported (Merlo et al., 2013). There was only one item (18) that would have led to a marginal improvement of internal consistency if removed: “I have almost caused an accident because of my cell phone use”. A possible explanation may lie in a slight ambiguity of wording, as the word “almost” is open to interpretation, and the word “accident” is not further specified. For example, using a mobile phone while driving a car is well-known to carry a high risk, so some people might equate using it while driving with almost causing an accident, whereas others may think of nearly bumping into someone while using a phone. However, since this item affects the quality of the test only to a minute degree, changing it must be weighed against modifying an established scale.

The present study was the first to investigate the questionnaire’s retest reliability, and it demonstrated a very good 14-day retest reliability of r_tt_ = 0.87. This indicates good psychometric characteristics of the scale and also suggests a reasonable stability of the measured construct.

Considering indicators of validity, an EFA with half of the sample reproduced the one-factor solution of the English original, but with 35.58% the explained variance was lower than in the English original (49.05%). With the other half of the sample, CFAs were calculated, testing three one-factor models, varying the covariance allowed. The best fit was shown by the model, in which the items 1 and 2, 3 and 14, 5 and 12, 8 and 20, 15 and 19, and 17 and 18 were allowed to covary. The decisions were based on the item content: Items 1 and 2 both refer to the satisfaction experienced as a function of time using the mobile phone. Items 3 and 14 both ask for consequences of a reduction of mobile phone use. Items 5 and 12 refer to avoidance of other tasks by using the mobile phone. Items 6 and 7 directly ask whether the person finds they are spending too much time using the mobile phone. Items 8 and 20 deal with using the mobile phone in the face of social pressure to the contrary. Items 15 and 19 focus on negative consequences of the mobile phone use that the person experienced and lastly, items 17 and 18 refer to dangerous situations occurring through the mobile phone use. For items 4, 10, 11 and 13 there seemed to be no a priori reason to consider that they may covary. The resulting CFA provides the best fit of the tested models as evidenced by the smallest AIC, and an acceptable SRMR and X2/df ratio. However, the RMSEA and the CFI, though the best ones of the models tested, remain unsatisfactory and future studies should investigate the factor structure further.

The high positive correlation with the MPPUS (*r* = 0.87), self-rated problematic user patterns (*r* = 0.65), and self-reported time of mobile phone use per day (*r* = 0.50) indicate good construct validity. For the item “I sometimes think that I might be ‘addicted’ to my cell phone”, (Merlo et al., 2013) found a correlation with the PUMP of *r* = 0.73, which is comparable to our slightly lower correlation of self-rated problematic user patterns with the PUMP-D, *r* = 0.65 (though not the same: z = 2.15; *p* = .016). The differences may be accounted for by the slightly different way in which the questions were phrased or differences in the sample investigated.

Our findings also indicate that problematic mobile phone use may be a relevant issue in Germany, as nearly one-third of the participants in our study rated their own user habits as rather problematic or problematic. The negative correlation of both the PUMP-D and the self-rating of problematic user patterns with the SES indicated a lower self-esteem in people with a higher problematic mobile phone use. The positive correlation of both the PUMP-D and the self-rating of problematic user patterns with the CES-D-10 fit well with the already existing studies (Demirci et al., 2015, Harwood et al., 2014) demonstrating an association of depressive symptoms and excessive mobile phone use.

The PUMP scale is not limited to any specific use of the mobile phone: we do not know whether a person spends too much time on social networks, games, or music applications rendering “problematic use of mobile phones” and “problematic use of an application” (e.g. Bergen Facebook Addiction Scale [Andreassen, Torsheim, Brunborg, & Pallesen, 2012]) indistinguishable. However, such a distinction is, in general, very difficult because of the rapid technological progress. Barnes (Barnes, Pressey, & Scornavacca, 2019) examined this topic and found in a comparison of questionnaire data higher scores for “mobile phone addiction” than for “addiction to social networks” and concluded “mobile phone addiction” is greater than “addiction to social networks”. This effect may be explained by the multi-faceted functionality of the mobile phone (Pearson & Hussain, 2015). This supports the use of questionnaires relating to the all-encompassing use of mobile phones, like the PUMP scale, but further validating research will be necessary.

There are a few limitations with regard to the interpretation of our results. All data are based on a convenience sample providing cross-sectional self-reports. Most of the participants had a young age (in the mean 27.8 years old (±11.2)) so this may not be representative for the general population.

## 5 Conclusion

The present study has established that the German version of the PUMP scale (PUMP-D)– as a brief instrument to assess problematic mobile phone use in German samples – has good psychometric properties, corroborating those reported for the original scale. Future research should investigate the factor structure further.

## CRediT authorship contribution statement

**Katharina Graben:** Data curation, Writing - original draft, Visualization. **Bettina K. Doering:** Conceptualization, Writing - review & editing. **Franziska Jeromin:** Conceptualization. **Antonia Barke:** Conceptualization, Project administration, Supervision, Writing - review & editing.

## Declaration of Competing Interest

The authors declare that they have no known competing financial interests or personal relationships that could have appeared to influence the work reported in this paper.
